# Evaluation of serum CEA, CYFRA21-1 and CA125 for the early detection of colorectal cancer using longitudinal preclinical samples

**DOI:** 10.1038/bjc.2015.202

**Published:** 2015-06-02

**Authors:** D S Thomas, E-O Fourkala, S Apostolidou, R Gunu, A Ryan, I Jacobs, U Menon, W Alderton, A Gentry-Maharaj, J F Timms

**Affiliations:** 1 Women's Cancer, Institute for Women's Health, University College London, Gower Street, London WC1E 6BT, UK; 2 Faculty of Medical and Human Sciences, 1.018 Core Technology Facility, University of Manchester, Grafton Street Manchester M13 9NT, UK; 3 Abcodia Ltd, The Network Building, 97 Tottenham Court Road, London W1T 4TP, UK

**Keywords:** colorectal cancer, preclinical serum biomarkers, UKCTOCS, CEA, CYFRA21-1

## Abstract

**Background:**

Blood-borne biomarkers for early detection of colorectal cancer (CRC) could markedly increase screening uptake. The aim of this study was to evaluate serum carcinoembryonic antigen (CEA), CYFRA21-1 and CA125 for the early detection of CRC in an asymptomatic cohort.

**Methods:**

This nested case–control study within UKCTOCS used 381 serial serum samples from 40 women subsequently diagnosed with CRC, 20 women subsequently diagnosed with benign disease and 40 matched non-cancer controls with three to four samples per subject taken annually up to 4 years before diagnosis. CEA, CYFRA21-1 and CA125 were measured using validated assays and performance of markers evaluated for different pre-diagnosis time groups.

**Results:**

CEA levels increased towards diagnosis in a third of all cases (half of late-stage cases), whereas longitudinal profiles were static in both benign and non-cancer controls. At a threshold of >5 ng ml^−1^ the sensitivities for detecting CRC up to 1 and 4 years before clinical presentation were 25% and 13%, respectively, at 95% specificity. At a threshold of >2.5 ng ml^−1^, sensitivities were 57.5% and 38.4%, respectively, with specificities of 81% and 83.5%. CYFRA21-1 and CA125 had no utility as screening markers and did not enhance CEA performance when used in combination. CEA gave average lead times of 17–24 months for test-positive cases.

**Conclusions:**

CEA is elevated in a significant proportion of individuals with preclinical CRC, but would not be useful alone as a screening tool. This work sets a baseline from which to develop panels of biomarkers which combine CEA for improved early detection of CRC.

Colorectal cancer (CRC) is a major burden worldwide. In the United Kingdom alone, there were 40 695 new cases and 15 708 mortalities in 2010 (CRUK, 2014). Development of a colorectal tumour to the point of metastasis, often incurable, is a lengthy period that proceeds through a pre-malignant stage, where simple polypectomy is curative, and an early, localised malignant stage that is treatable. Survival rates are more favourable when detected earlier; 93, 77, 48 and 7% of those diagnosed at stages I to IV, respectively, survive 5 years (NCIN, 2010). Early detection of CRC is therefore crucial to reducing mortality from the disease.

Advanced polyps and cancers bleed intermittently and the faecal occult blood test (FOBT) is used to detect trace amounts of haemoglobin in stool samples. Biennial FOBT-based screening, which was implemented in the National Health Service (NHS) Bowel Cancer Screening Programme (NHSBCSP) in the United Kingdom, is associated with a 15% reduction in mortality and a stage shift towards earlier detection (Hewitson *et al*, 2007; Gill *et al*, 2012; Logan *et al*, 2012). Other trials have reported mortality reductions of 15%–33% using FOBT-based screening (Mandel *et al*, 1993; Selby *et al*, 1993; Hardcastle *et al*, 1996; Kronborg *et al*, 1996; Lindholm *et al*, 2008). The potential benefits are compromised by the limited sensitivity (13–50%) of the FOBT for detection in asymptomatic cohorts (Allison *et al*, 1990; Imperiale *et al*, 2004) and poor uptake (52%) since implementation (Logan *et al*, 2012). Flexible sigmoidoscopy is the other first line screening test (followed by colonoscopy if positive) that affords greater sensitivity over FOBT, detecting 70–80% of advanced neoplasms of the colorectum (Whitlock *et al*, 2008). It has proven to be efficacious for screening (Atkin *et al*, 2010; Holme *et al*, 2013; Schoen *et al*, 2012), although cannot be used to detect the ∼40% of tumours that develop in the proximal colon (Whitlock *et al*, 2008). Screening with gold-standard diagnostic colonoscopy is advocated for high-risk groups in the United Kingdom (Cairns *et al*, 2010) and sporadic cancer in the United States (Levin *et al*, 2008). However, compliance rates for these invasive tests are low (Robb *et al*, 2010; Taylor *et al*, 2011).

Blood tests are routinely used for biomarker determination and are widely accepted, and it is reasoned that the transition to an initial blood tumour marker test for CRC screening would improve uptake due its less invasive nature than either FOBT or flexible sigmoidoscopy. However, no serum tumour markers have approved screening utility for CRC (Locker *et al*, 2006; Duffy *et al*, 2014), and although a recently evaluated plasma septin 9 (SEPT9) DNA methylation test (Epi proColon) holds some promise (Church *et al*, 2013; Johnson *et al*, 2014), increased uptake (*vs* faecal testing) in non-compliant populations needs to be demonstrated. Poor clinical translation may be, in part, due to the lack of established biorepositories with extended patient follow-up that can yield preclinical samples drawn from asymptomatic individuals who eventually developed cancer. Biomarker testing in such samples is likely to improve validity and confidence in identifying markers with screening utility in the absence of clinical stage confounders (Pepe *et al*, 2008; Buchen, 2011).

Carcinoembryonic antigen (CEA) is the most routinely used colorectal tumour marker, and is recommended by the National Academy of Clinical Biochemistry and American Society of Clinical Oncology for prognosis, monitoring response to treatment and for detecting metastatic disease and disease recurrence (Locker *et al*, 2006; Duffy *et al*, 2014). However, serum CEA has limited sensitivity for screening in asymptomatic people. CEA testing on 46 preclinical cases (29 early stage/17 advanced stage) provided a lead time of up to 2 years in 30% of future CRCs at a cutoff threshold that correctly identified 99% of controls (Palmqvist *et al*, 2003). In another study, elevated CEA conferred a lead time of up to 7 months in 19% of 32 (17 early stage/15 advanced stage) preclinical cases (Ladd *et al*, 2012). Both studies, however, involved the use of a single cross-sectional sample and were limited to a maximum of a 2-year lead time. The circulating cytokeratin 19 fragment, CYFRA21-1, has been demonstrated as a useful biomarker in several malignancies, notably lung, urinary bladder and head and neck cancers (Barak *et al*, 2004). The diagnostic performance of CYFRA21-1 for CRC has been assessed in two studies (Wild *et al*, 2010; Lee, 2013), although its potential for screening using preclinical samples has not been evaluated.

Herein, we present a nested case–control study within the UK Collaborative Trial for Ovarian Cancer Screening (UKCTOCS); a multi-centre randomised controlled trial that aims to inform on the viability of an ovarian cancer screening programme in the United Kingdom (Menon *et al*, 2008, 2009). The UKCTOCS biorepository includes samples from 50 640 women randomised to the multi-modal arm, who donated serum annually for up to 11 years for ovarian cancer screening using cancer antigen 125 (CA125) levels. The present study aims to evaluate the performance of CEA, CYFRA21-1 and CA125 for the early detection of CRC in an asymptomatic cohort by profiling tumour marker levels in four annual longitudinal serum samples collected from women who subsequently developed CRC, benign neoplasms of the colorectum or remained cancer free. We also wanted to address the reported link between smoking and raised serum CEA levels (Alexander *et al*, 1976; Chevinsky, 1991).

## Materials and methods

### Ethical approval

The present study was approved by the NHS National Research Ethics Service (REC 13/EM/0191). UKCTOCS participants gave informed written consent at recruitment for the use of their medical notes and serum in secondary and/or commercial studies. Ethical approval for UKCTOCS was granted by the UK North West Medical Research and Ethics Committee (MREC 00/8/34).

### Case identification, confirmation and characterisation

UKCTOCS participants were post-menopausal women aged 50–74, who had no active malignancy at recruitment (Menon *et al*, 2008). Notifications of women subsequently diagnosed with CRC were retrieved by querying the Health and Social Care Information Centre cancer and death registries and Hospital Episode Statistics (HES) data with the International Classification of Diseases (ICD-10) codes pertaining to malignant neoplasms of the colon (C18, excluding appendix (C18.1)), the rectosigmoid junction (C19) and rectum (C20). Cancer notifications were also received via self-reported data completed 3.5 years post randomisation to the UKCTOCS. CRC notifications were confirmed and characterised by postal questionnaire sent to treating clinicians (consultant, or General Practitioner if details not provided by the volunteer), which was designed to ascertain clinical and histological data on diagnosed cases (date of diagnosis, primary site, stage, grade, morphology and treatment). Benign neoplasms of the colon and rectum (D12), excluding those of the appendix (D12.1), anus and anal canal (D12.9) were identified through HES (England only).

### Study set

The study set consisted of longitudinal preclinical samples collected 0–4 years before the eventual diagnosis of colorectal adenocarcinoma in 40 women (20 early stage, defined as Dukes' A/B and 20 late stage, defined as Dukes' C/D), or benign neoplasms of the colon and rectum in 20 women, and matched samples from 40 control women; 20 matched to the early stage and 20 matched to the late-stage adenocarcinoma cases; see Supplementary Data, Supplementary Table S1 for clinical and histological data, Supplementary Table S2 for the number of samples associated with each time and clinical group). Benign cases did not develop any type of malignancy during the study period and had no previous diagnoses of any type of malignancy according to HES records. Likewise, non-cancer controls did not develop, and had no previous record of any type of malignancy according to the cancer registry, HES, UKCTOCS or self-reported data, or any diagnosis of a benign neoplasm of the colorectum according to HES. Benign cases were matched 1:1 with early-stage adenocarcinoma cases by age at sample donation (±5 years) and collection centre (same, excluding six cases in trial centres in Northern Ireland and Wales, which were matched to the nearest trial centre in England). Non-cancer controls were individually matched 1:1 to early and late-stage cases by trial centre, age at final sample draw (±5 years) and date of sample draw (same day for 0–1 year preclinical sample and ±4 months for 3–4 years preclinical sample). Baseline characteristics, ethnicity, current hormone replacement therapy (HRT) use, oral contraceptive pill (OCP) use, OCP use duration, ever smokers, age at randomisation, body mass index (BMI) and age at last period were taken from the UKCTOCS recruitment questionnaire (Fourkala *et al*, 2014) and are shown for all study subjects in Table 1.

### Serum marker determinations

Blood was collected and serum prepared according to a standardised protocol within UKCTOCS (Menon *et al*, 2009) and then shipped frozen to a cryo-repository for long-term storage in liquid nitrogen. For the study, samples were retrieved and shipped to the laboratory on dry ice and thawed at 4 °C for aliquoting before carrying out assays. Serum CEA and CYFRA21-1 levels were determined using the Cobas immunoassays and platform (Roche Diagnostics, Burgess Hill, UK) with relevant calibrator set and PreciControl tumour marker standards for quality control (Roche Diagnostics). All marker determinations were carried out by a single experienced researcher (RG) who was blinded to all information regarding the sample and UKCTOCS volunteer. The present study made use of CA125 levels determined previously for the UKCTOCS.

## Results

### Study set characteristics

The study set comprised of 381 longitudinal serum samples from 100 women for CEA and CYFRA21-1 testing and 456 CA125 measurements from the same women (Supplementary Data, Supplementary Table S2). These samples were drawn from groups of 20 early- and late-stage CRC cases and matched benign and non-cancer controls. There was no significant difference between these groups in terms of their ethnicity, the number of current HRT and OCP users, ever smokers, OCP use duration, age at randomisation, BMI and age at last period (Table 1). Furthermore, there was no significant difference between the groups in terms of the time from sample draw to centrifugation (data not shown) with a median time to spin of 21.7 h (IQR 20.0–23.6 h). Thus, differences in time to spin could not account for any differences in serum analyte levels between groups.

### Longitudinal profiles

**Figure 1:**
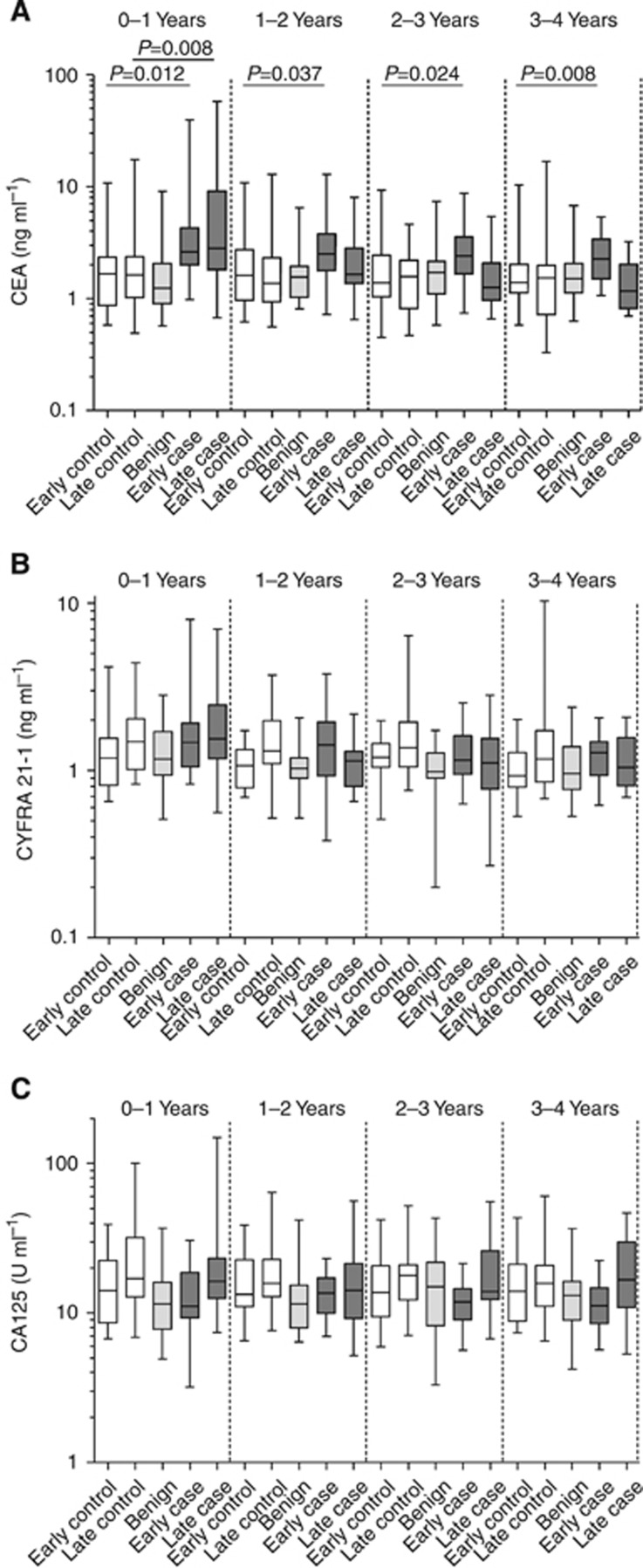
**Serum measurements.** Serum measurements for (**A**) CEA, (**B**) CYFRA21-1 and (**C**) CA125. Box and whiskers denote the 25th/50th/75th percentiles and minimum and maximum values, respectively. Early case refers to Dukes' stages A and B colorectal adenocarcinomas. Late case refers to Dukes' stages C and D colorectal adenocarcinomas. Early and late controls are the corresponding matched non-cancer controls. Date of diagnosis for controls corresponds with that of their matched case.

**Figure 2:**
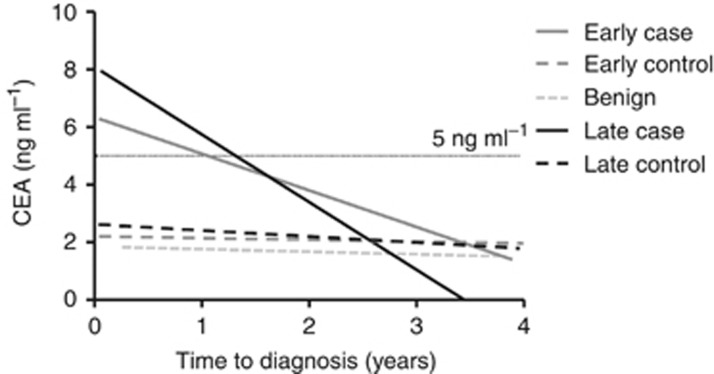
**Linear regression longitudinal CEA profiles in cases and controls.** The linear regression models with *R*^2^=0.072 and *R*^2^=0.133 for early- and late-stage cases, respectively, significantly deviated from zero (*P*<0.05). A CEA threshold of >5 ng ml^−1^ gave a lead time benefit of 1.07 and 1.32 years for detection of early- and late-stage cancers, respectively. At a threshold of >2.5 ng ml^−1^, lead times were 3.02 and 2.38 years, respectively.

The longitudinal behaviour of serum CEA, CYFRA21-1 and CA125 in the lead up to cancer and benign neoplasm diagnosis and in matched non-cancer controls was assessed. Graphical representations of these determinations stratified according to clinical group and time to diagnosis group are shown in Figure 1 with median values for groups presented in Table 2. Elevated levels of CEA in cases (all stages) compared with non-cancer controls were significant up to 2 years before diagnosis (*P*<0.05) and could also discriminate cancers from benign neoplasms (data not shown). Notably, CEA levels in early-stage cases were significantly elevated >2 years compared with late-stage cases (*P*<0.05). This difference could be visualised with a linear regression model (Figure 2). Examination of individual longitudinal profiles (Supplementary Data, Supplementary Figure S1) showed CEA levels to be rising towards diagnosis only in cancer cases and to be relatively static in benign and non-cancer controls at each annual blood draw. Elevation of CEA towards diagnosis was apparent in 3/20 early-stage and 10/20 late-stage cancer cases. Several subjects (three CRC, one benign and three non-cancer controls) had high CEA levels (>4 ng ml^−1^) that were static across the time course.

CYFRA21-1 levels were not significantly different (*P*>0.05) between cases and controls for any of the time groups examined (Table 2) and only weak and non-significant correlations were observed between CYFRA21-1 levels and time to diagnosis (*R*^2^=0.115 for early stage; *R*^2^=0.094 for late stage). CYFRA21-1 levels rose towards diagnosis in 17 of the 40 (42.5%) CRC cases, but also in 17 of the 60 (28.3%) benign and non-cancer controls, although levels were rarely above the clinical threshold of 3.3 ng ml^−1^ (Molina *et al*, 1994; Rastel *et al*, 1994; data not shown). Changing CYFRA21-1 levels were not attributable to time in storage or time to spin. CA125 levels did not differ significantly between cases and controls for any of the time groups, although levels were significantly higher in the late stage *vs* early-stage case samples taken 2–3 and 3–4 years before diagnosis.

### Biomarker performance

CEA and CYFRA21-1 were assessed individually and in combination for their ability to discriminate all cases from benign and non-cancer controls using different cutoff values (Supplementary Data, Supplementary Table S3A). CA125 was not examined in combination. At the commonly used threshold of >5 ng ml^−1^, CEA had a sensitivity and specificity of 25% and 95% at 0–1 years, 14% and 92% at 1–2 years, 11% and 98% at 2–3 years, 3% and 93% at 3–4 years and 13% and 94% 0–4 years to diagnosis. Specificity values changed little when only the cases of benign neoplasms were considered (Supplementary Table S3B). At a lower cutoff value of 2.5 ng ml^−1^, the sensitivity and specificity were 57.5% and 81% at 0–1 years, 37.8% and 87.9% at 1–2 years, 30.6% and 83.6% at 2–3 years, 26.3% and 88.3% at 3–4 years and 38.4% and 83.5% 0–4 years (Supplementary Table S3A). CYFRA21-1 at a cutoff threshold of 3.3 ng ml^−1^ had encouraging specificities (all >96%), but only detected 4 and 1 out of the 40 cases in the 0–1 and 1–2 year time groups, respectively, and detected no preclinical benign neoplasms. At a 2 ng ml^−1^ threshold, the sensitivity for CYFRA21-1 was 14.6% at a specificity of 90% when all samples were considered. Simple combination ‘OR' models showed some improvement in sensitivity, but at significant cost to specificity (Supplementary Table S3). ROC curve analysis confirmed that CEA was able to differentiate all cases from controls up to 2 years before diagnosis, with superior and significant areas under the curve demonstrated for early *vs* late-stage cases beyond 2 years (Table 2). The poor performance of CYFRA21-1 as an early biomarker of CRC was further highlighted by insignificant areas under the curve. Neither CEA nor CYFRA21-1 could significantly discriminate benign cases from non-cancer controls.

Lead time estimates were calculated by averaging the earliest time point of detection for CEA test-positive cases at the 5 and 2.5 ng ml^−1^ thresholds. Mean lead time was 16.9 months (median 17.9 months; IQR 4.9–26.3) using >5 ng ml^−1^ and 24.1 months (median 23.0 months; IQR 7.3–39.9) using >2.5 ng ml^−1^. By comparison, linear regression models (Figure 2) estimated a lead time of 12.8 and 15.8 months at >5 ng ml^−1^ for detecting early- and late-stage cancers, respectively, whereas at >2.5 ng ml^−1^, the lead time was 36.2 and 28.6 months, respectively.

### Smoking and CEA levels

To address the link between smoking and raised CEA levels, we combined smoking data (ever/never) provided by UKCTOCS women at 3.5 years post randomisation and CEA levels determined at 3-4 years pre-diagnosis for 78 of the 100 study subjects. Using this subset of data, there was a significant positive association between elevated CEA (>5 ng ml^−1^) and ever smoking (*P*=0.042), although this significance was lost when the lower threshold (>2.5 ng ml^−1^) was used. Using the whole data set (304 CEA data points from 80 respondents), the association between smoking and CEA level was significant at both thresholds (>5 ng ml^−1^, *P*=0.031 and >2.5 ng ml^−1^, *P*=0.0047). Furthermore, of the three out of the seven subjects with consistently elevated CEA who had responded about smoking, all three were ever smokers.

## Discussion

To our knowledge, this is the first study to examine the serum levels of CEA, CYFRA21-1 and CA125 in longitudinal samples taken before the diagnosis of CRC. CEA level increased towards diagnosis in 32.5% of all cases and was raised above 2.5 ng ml^−1^ 3–4 years before diagnosis in 26.3% of cases. Longitudinal CEA levels did not change significantly over time in any of the benign cases or non-cancer controls. This suggests that only a subset of colorectal adenocarcinomas produce an elevation in serum CEA, and that this is specific to the malignant phenotype (Hammarstrom, 1999). Rising CEA towards diagnosis was more frequent in late-stage tumours, as reported previously (Wanebo *et al*, 1978), but did not correlate with grade (data not shown), as suggested previously (Goslin *et al*, 1981). Although we confirmed that ever smokers were significantly more likely to have elevated CEA levels, only two cases with rising CEA profiles were ever smokers. Indeed, ever smokers tended to have elevated, but static longitudinal CEA profiles, and occurred equally in both cases and controls with only a small impact on specificity.

Using a threshold of >5 ng ml^−1^, CEA had a sensitivity of 25% at 95% specificity up to 1 year before clinical diagnosis, and 13% sensitivity up to 4 years. In a screening programme, this would result in one in four cancers being detected up to 1 year before clinical presentation and 5 out of 100 ‘healthy' attendants being referred for colonoscopy unnecessarily. Lowering the threshold to 2.5 ng ml^−1^ would result in 32.5% additional cancers being detected up to 1 year before clinical presentation (25.4% up to 4 years), but may cause an unacceptably high proportion (14%) of false-positives (10.5% for up to 4 years) requiring unnecessary colonoscopy investigations. These findings are in line with other studies examining CEA in cross-sectional pre-diagnosis samples (Palmqvist *et al*, 2003; Ladd *et al*, 2012), and we conclude therefore that on its own, serum CEA would have little use in screening asymptomatic, average risk populations. Despite this, we report respectable lead times in women with elevated CEA, indicating its potential as a first line test for early detection, particularly if it were to be combined with other markers, or used in a longitudinal algorithm, to increase performance. Although CEA may be superior to the guaiac FOBT (Allison *et al*, 1990, 1996; Ransohoff and Lang, 1997; Imperiale *et al*, 2004), it appears inferior to Cologuard (a faecal test combining haemoglobin protein, NDRG4 and BMP3 gene promoter hypermethylation, seven *KRAS* gene point mutations and *β*-actin DNA as a normalisation marker), the faecal immunochemical test (a more precise version of the FOBT for detecting haemoglobin) and Epi proColon (plasma SEPT9 DNA methylation), which have been evaluated in large prospective trials (Guittet *et al*, 2007; Church *et al*, 2013; Raginel *et al*, 2013; Johnson *et al*, 2014; Lee *et al*, 2014; Imperiale *et al*, 2014a, 2014b). However, we highlight the fact that the faecal-based tests have relatively poor acceptance to the general population compared with blood-based tests due to a general aversion to faecal sampling. Importantly, the performance of these tests far in advance of diagnosis, and the lead time benefits afforded, have yet to be determined. Thus, CEA may still have some utility for the earlier detection of CRC if used in combination with a more sensitive marker. Such markers should complement CEA, detecting those cancers that were not positive for CEA, with TIMP1, VEGF, sCD26 and PKM2 showing some promise.

Despite numerous reports of CYFRA21-1 as a specific marker of multiple malignancies and our evidence of its elevation towards diagnosis in some cases, its performance as a screening marker was very poor and it did not add to CEA when used in combination. We conclude that serum CYFRA21-1 cannot be used as an early marker of CRC and would have limited diagnostic use. Similarly, and perhaps as expected, CA125 also proved to be a poor biomarker of preclinical CRC.

Our study has several limitations. First, only post-menopausal women were studied, and although this reflects the timing of diagnosis of most CRCs it may not reflect the utility of CEA as a colorectal tumour marker in the overall population. Given that incidence rates are around 1.7 times higher in males *vs* females in this age group, then 63% of the population would not be represented. Second, only relatively small numbers of cases and controls were examined, although they were carefully matched and with longitudinal samples selected up to 4 years prior to diagnosis. Third, the smoking status of our study cohort was not complete and was restricted to dichotomous data (ever/never), limiting our evaluation of its impact on test performance. However, the key strength of the study is that highly characterised preclinical, longitudinal samples were investigated, allowing an objective assessment of how serological markers change during disease progression. We conclude that in line with previous studies, CEA alone cannot be used for CRC screening in asymptomatic populations. Despite this, our work lays the groundwork for building and assessing longitudinal algorithms for CRC screening and combining promising new candidate biomarkers with CEA to improve performance.

## Figures and Tables

**Table 1:** Baseline characteristics of study set

**Baseline characteristic**	**Control (matched to early-stage case),** ***n*****=20**	**Early-stage case (Duke's A/B),** ***n*****=20**	**Benign,** ***n*****=20**	**Control (matched to late-stage case),** ***n*****=20**	**Late-stage case (Duke's C/D),** ***n*****=20**	***P*****-value**
	**Number (%)**					***χ***^**2**^**-test**
Ethnicity (*n*)						0.978
White	19 (95%)	19 (95%)	18 (90%)	20 (100%)	19 (95%)	
Black	0 (0%)	0 (0%)	0 (0%)	0 (0%)	0 (0%)	
Other	1 (5%)	1 (5%)	2 (10%)	0 (0%)	1 (5%)	
Current HRT users (*n*)	4 (20%)	5 (25%)	5 (5%)	3 (15%)	3 (15%)	0.87
Ever OCP users (*n*)	7 (35%)	10 (50%)	12 (60%)	10 (50%)	6 (30%)	0.303
Ever smokers (*n*)	7 (35%)	7 (35%)	8 (40%)	5 (25%)	2 (10%)	0.161
	Mean (s.d.)					ANOVA
Age at randomisation (years)	61.3 (5.2)	62.1 (5.5)	61.5 (6.1)	62.5 (6.0)	62.7 (6.0)	0.913
BMI (kg m^−2^)	26.0 (4.2)	25.8 (5.3)	27.4 (5.4)	26.6 (5.1)	25.3 (3.5)	0.667
Age at last period (years)	49.9 (7.5)	49.5 (4.9)	47.7 (6.3)	49.9 (5.3)	49.5 (4.7)	0.742
	Median (IQR)					Kruskal–Wallis
OCP duration in users (years)	10.0 (4)	3.0 (11.8)	5.5 (8.0)	5.0 (6.0)	5.5 (16.6)	0.307

Abbreviations: ANOVA=analysis of variance; BMI=body mass index; HRT=hormone replacement therapy; IQR=interquartile range; OCP=oral contraceptive pill.

Early case refers to Dukes' stages A and B colorectal adenocarcinomas. Late case refers to Dukes' stages C and D colorectal adenocarcinomas. Early and late controls are the corresponding matched non-cancer controls.

**Table 2:** Serum CEA and CYFRA21-1 by time to diagnosis group and clinical group

**Years to diagnosis**		**CEA**				**CYFRA21-1**			
**Group**	**Mean (years)**	**Median (ng ml**^**−1**^)	***P*****-value** ***vs*** **Ctrl**	**AUC**	***P*****-value AUC**	**Median (ng ml**^**−1**^)	***P*****-value** ***vs*** **Ctrl**	**AUC**	***P*****-value AUC**
**All stages**									
0–1	0.48	2.7	0.0002	0.74	<0.001	1.47	NS	0.6	NS
1–2	1.49	2.03	0.042	0.64	0.042	1.18	NS	0.51	NS
2–3	2.46	1.83	NS	0.61	NS	1.15	NS	0.58	NS
3–4	3.48	1.66	NS	0.59	NS	1.13	NS	0.52	NS
**Early stage**									
0–1	0.48	2.61	0.012	0.73	0.011	1.47	NS	0.66	NS
1–2	1.55	2.51	0.037	0.7	0.036	1.42	NS	0.67	NS
2–3	2.48	2.4	0.024	0.71	0.023	1.15	NS	0.53	NS
3–4	3.57	2.27	0.008	0.76	0.007	1.28	NS	0.64	NS
**Late stage**									
0–1	0.48	2.81	0.008	0.75	0.007	1.54	NS	0.56	NS
1–2	1.44	1.65	NS	0.59	NS	1.14	NS	0.65	NS
2–3	2.43	1.26	NS	0.51	NS	1.11	NS	0.67	NS
3–4	3.4	1.17	NS	0.51	NS	1.04	NS	0.6	NS
**Benign**									
0–1	0.59	1.25	NS	0.58	NS	1.17	NS	0.54	NS
1–2	1.58	1.55	NS	0.54	NS	1.03	NS	0.54	NS
2–-3	2.49	1.72	NS	0.5	NS	0.99	NS	0.66	NS
3–4	3.53	1.52	NS	0.54	NS	0.96	NS	0.51	NS
**Control early**									
0–1	0.48	1.67	—	—	—	1.18	—	—	—
1–2	1.44	1.61	—	—	—	1.07	—	—	—
2–3	2.43	1.39	—	—	—	1.2	—	—	—
3–4	3.4	1.4	—	—	—	0.93	—	—	—
**Control late**									
0–1	0.48	1.62	—	—	—	1.49	—	—	—
1–2	1.44	1.37	—	—	—	1.31	—	—	—
2–3	2.43	1.58	—	—	—	1.37	—	—	—
3–4	3.4	1.53	—	—	—	1.17	—	—	—

Abbreviations: AUC=area under the curve; CEA=carcinoembryonic antigen; Ctrl=control; NS=non-significant.

Median values and *P*-values (*vs* controls) and areas under the ROC curve (AUC) and associated *P*-value are given for each group and comparison. Refer to Supplementary Table S2 for sample numbers in each group.

## Notes

UM and IJ have financial interest through UCL Business and Abcodia Ltd in the third-party exploitation of clinical trials biobanks which have been developed through research at UCL. The remaining authors declare no conflict of interest.
