# Supplementary material for: Evaluation of serum CEA, CYFRA21-1 and CA125 for the early detection of colorectal cancer using longitudinal preclinical samples
Source: Br J Cancer. 2015 Jun 2;113(2):268–74. doi: 10.1038/bjc.2015.202 (PMC4506388; doi:10.1038/bjc.2015.202)
Supplement: Supplementary Information [file bjc2015202x1.pdf]

Supplementary Data

Table S1 Clinico-pathological data associated with malignant and benign cases

	Early stage (n=20)	Late stage (n=20)	Benign (n=20)
Primary site			
Caecum	3 (15%)	4 (20%)	1 (5%)
Ascending colon	6 (30%)	2 (10%)	0 (0%)
Transverse colon	0 (0%)	3 (15%)	3 (15%)
Splenic flexure	1 (5%)	0 (0%)	0 (0%)
Descending colon	1 (5%)	0 (0%)	2 (10%)
Sigmoid colon	5 (25%)	5 (25%)	8 (40%)
Rectosigmoid	0 (0%)	0 (0%)	1 (5%)
Rectum	3 (15%)	6 (30%)	2 (10%)
Undetermined	1 (5%)	0 (0%)	3 (15%)
Standardised stage			
A	7 (35%)	0 (0%)	NA
B	13 (65%)	0 (0%)	NA
C	0 (0%)	14 (70%)	NA
D	0 (0%)	6 (30%)	NA
Grade			
I	1 (5%)	2 (10%)	NA
II	12 (60%)	12 (60%)	NA
III	3 (15%)	2 (10%)	NA
Unknown	4 (20%)	4 (20%)	
Morphology			
Adenocarcinoma	20 (100%)	20 (100%)	NA

Table S2 Study group sample sizes for CEA, CYFRA21-1 and CA125 determinations.

	CEA and CYFRA21-1/CA125				
Years to diagnosis	Early control	Early case	Benign	Late control	Late case
0-1	20/24	20/23	18/20	20/30	20/29
1-2	19/24	18/23	20/20	18/21	19/22
2-3	20/20	19/23	18/24	17/22	17/20
3-4	20/20	18/20	20/22	20/22	20/27

Table S3 Tumour marker performance according to different cut-off thresholds (ng/mL) and pre-diagnosis time group. 'CEA or CYFRA' refers to a combined panel where an elevated level of either marker is considered positive. (A) Cancer cases versus non-cancer controls + benigns; (B) Cancer cases versus benign only. Green shading for sensitivity and red shading for specificity is scaled from highest to lowest value in each table.

(A)

	Time group	Number/Parameter	CEA >5 ng/mL	CYFRA >3.3 ng/mL	CEA or CYFRA	CEA >3 ng/mL	CYFRA >2 ng/mL	CEA or CYFRA	CEA >2.5 ng/mL	CYFRA >1.5 ng/mL	CEA or CYFRA	CEA >2.5 ng/mL	CYFRA >3.3 ng/mL	CEA or CYFRA
All cases vs. Control + Benign	0-1 yr	Case positive (n)	10	4	12	16	11	20	23	20	31	23	4	24
		Case negative (n)	30	36	28	24	29	20	17	20	9	17	36	16
		Control positive (n)	3	2	5	8	9	17	11	21	30	11	2	13
		Control negative (n)	55	56	53	50	49	41	47	37	28	47	56	45
		Sensitivity (%)	25.0	10.0	30.0	40.0	27.5	50.0	57.5	50.0	77.5	57.5	10.0	60.0
		Specificity (%)	94.8	96.6	91.4	86.2	84.5	70.7	81.0	63.8	48.3	81.0	96.6	77.6
	1-2 yr	Case positive (n)	5	1	5	11	5	13	14	10	20	14	1	14
		Case negative (n)	32	36	32	26	32	24	23	27	17	23	36	23
		Control positive (n)	4	2	6	6	4	10	11	9	18	11	2	13
		Control negative (n)	90	92	87	87	87	79	80	75	63	80	92	77
		Sensitivity (%)	13.5	2.7	13.5	29.7	13.5	35.1	37.8	27.0	54.1	37.8	2.7	37.8
		Specificity (%)	95.7	97.9	93.5	93.5	95.6	88.8	87.9	89.3	77.8	87.9	97.9	85.6
	2-3 yr	Case positive (n)	4	0	4	9	3	10	11	11	18	11	0	11
		Case negative (n)	32	36	32	27	33	26	25	25	18	25	36	25
		Control positive (n)	2	1	3	4	4	8	9	12	19	9	1	10
		Control negative (n)	53	54	52	51	51	47	46	43	36	46	54	45
		Sensitivity (%)	11.1	0.0	11.1	25.0	8.3	27.8	30.6	30.6	50.0	30.6	0.0	30.6
		Specificity (%)	96.4	98.2	94.5	92.7	92.7	85.5	83.6	78.2	65.5	83.6	98.2	81.8
	3-4 yr	Case positive (n)	1	0	1	9	3	11	10	10	16	10	0	10
		Case negative (n)	37	38	37	29	35	27	28	28	22	28	38	28
		Control positive (n)	4	1	5	4	6	10	7	13	20	7	1	8
		Control negative (n)	56	59	55	56	54	50	53	47	40	53	59	52
		Sensitivity (%)	2.6	0.0	2.6	23.7	7.9	28.9	26.3	26.3	42.1	26.3	0.0	26.3
		Specificity (%)	93.3	98.3	91.7	93.3	90.0	83.3	88.3	78.3	66.7	88.3	98.3	86.7
	1-4 yr	Case positive (n)	10	1	10	29	11	34	35	31	54	35	1	35
		Case negative (n)	101	110	101	82	100	77	76	80	57	76	110	76
		Control positive (n)	10	4	14	14	14	28	27	34	57	27	4	31
		Control negative (n)	162	168	158	158	158	144	145	138	115	145	168	141
		Sensitivity (%)	9.0	0.9	9.0	26.1	9.9	30.6	31.5	27.9	48.6	31.5	0.9	31.5
		Specificity (%)	94.2	97.7	91.9	91.9	91.9	83.7	84.3	80.2	66.9	84.3	97.7	82.0
	0-4 yr	Case positive (n)	20	5	22	45	22	54	58	51	85	58	5	59
		Case negative (n)	131	146	129	106	129	97	93	100	66	93	146	92
		Control positive (n)	13	6	19	22	23	45	38	55	87	38	6	44
		Control negative (n)	217	224	211	208	207	185	192	175	143	192	224	186
		Sensitivity (%)	13.2	3.3	14.6	29.8	14.6	35.8	38.4	33.8	56.3	38.4	3.3	39.1
		Specificity (%)	94.3	97.4	91.7	90.4	90.0	80.4	83.5	76.1	62.2	83.5	97.4	80.9

(B)

	Time group	Number/Parameter	CEA >5 ng/mL	CYFRA >3.3 ng/mL	CEA or CYFRA	CEA >3 ng/mL	CYFRA >2 ng/mL	CEA or CYFRA	CEA >2.5 ng/mL	CYFRA >1.5 ng/mL	CEA or CYFRA	CEA >2.5 ng/mL	CYFRA >3.3 ng/mL	CEA or CYFRA
**All Cases vs. Benign**	0-1 yr	Case positive (n)	10	4	12	16	11	20	23	20	31	23	4	24
		Case negative (n)	30	36	28	24	29	20	17	20	9	17	36	16
		Control positive (n)	1	0	1	2	2	4	3	6	8	3	0	3
		Control negative (n)	17	18	17	16	16	14	15	12	10	15	18	15
		Sensitivity (%)	25.0	10.0	30.0	40.0	27.5	50.0	57.5	50.0	77.5	57.5	10.0	60.0
		Specificity (%)	94.4	100.0	94.4	88.9	88.9	77.8	83.3	66.7	55.6	83.3	100.0	83.3
	1-2 yr	Case positive (n)	5	1	5	11	5	13	14	10	20	14	1	14
		Case negative (n)	32	36	32	26	32	24	23	27	17	23	36	23
		Control positive (n)	1	0	1	2	1	3	2	2	4	2	0	2
		Control negative (n)	19	20	19	18	19	17	18	18	16	18	20	18
		Sensitivity (%)	13.5	2.7	13.5	29.7	13.5	35.1	37.8	27.0	54.1	37.8	2.7	37.8
		Specificity (%)	95.0	100.0	95.0	90.0	95.0	85.0	90.0	90.0	80.0	90.0	100.0	90.0
	2-3 yr	Case positive (n)	4	0	4	9	3	10	11	11	18	11	0	11
		Case negative (n)	32	36	32	27	33	26	25	25	18	25	36	25
		Control positive (n)	1	0	1	1	0	1	3	1	3	3	0	3
		Control negative (n)	17	18	17	17	18	17	15	17	15	15	18	15
		Sensitivity (%)	11.1	0.0	11.1	25.0	8.3	27.8	30.6	30.6	50.0	30.6	0.0	30.6
		Specificity (%)	94.4	100.0	94.4	94.4	100.0	94.4	83.3	94.4	83.3	83.3	100.0	83.3
	3-4 yr	Case positive (n)	1	0	1	9	3	11	10	10	16	10	0	10
		Case negative (n)	37	38	37	29	35	27	28	28	22	28	38	28
		Control positive (n)	1	0	1	1	3	4	2	3	5	2	0	2
		Control negative (n)	19	20	19	19	17	16	18	17	15	18	20	18
		Sensitivity (%)	2.6	0.0	2.6	23.7	7.9	28.9	26.3	26.3	42.1	26.3	0.0	26.3
		Specificity (%)	95.0	100.0	95.0	95.0	85.0	80.0	90.0	85.0	75.0	90.0	100.0	90.0
	1-4 yr	Case positive (n)	10	1	10	29	11	34	35	31	54	35	1	35
		Case negative (n)	101	110	101	82	100	77	76	80	57	76	110	76
		Control positive (n)	3	0	3	4	4	8	7	6	12	7	0	7
		Control negative (n)	55	58	55	54	54	50	51	52	46	51	58	51
		Sensitivity (%)	9.0	0.9	9.0	26.1	9.9	30.6	31.5	27.9	48.6	31.5	0.9	31.5
		Specificity (%)	94.8	100.0	94.8	93.1	93.1	86.2	87.9	89.7	79.3	87.9	100.0	87.9
	0-4 yr	Case positive (n)	20	5	22	45	22	54	58	51	85	58	5	59
		Case negative (n)	131	146	129	106	129	97	93	100	66	93	146	92
		Control positive (n)	4	0	4	6	6	12	10	12	20	10	0	10
		Control negative (n)	72	76	72	70	70	64	66	64	56	66	76	66
		Sensitivity (%)	13.2	3.3	14.6	29.8	14.6	35.8	38.4	33.8	56.3	38.4	3.3	39.1
		Specificity (%)	94.7	100.0	94.7	92.1	92.1	84.2	86.8	84.2	73.7	86.8	100.0	86.8

Figure S1 Serum CEA longitudinal profiles in individual case control samples. (A) Early stage cases; (B) Late stage cases. Horizontal line represents 5 ng/mL CEA.

(A)

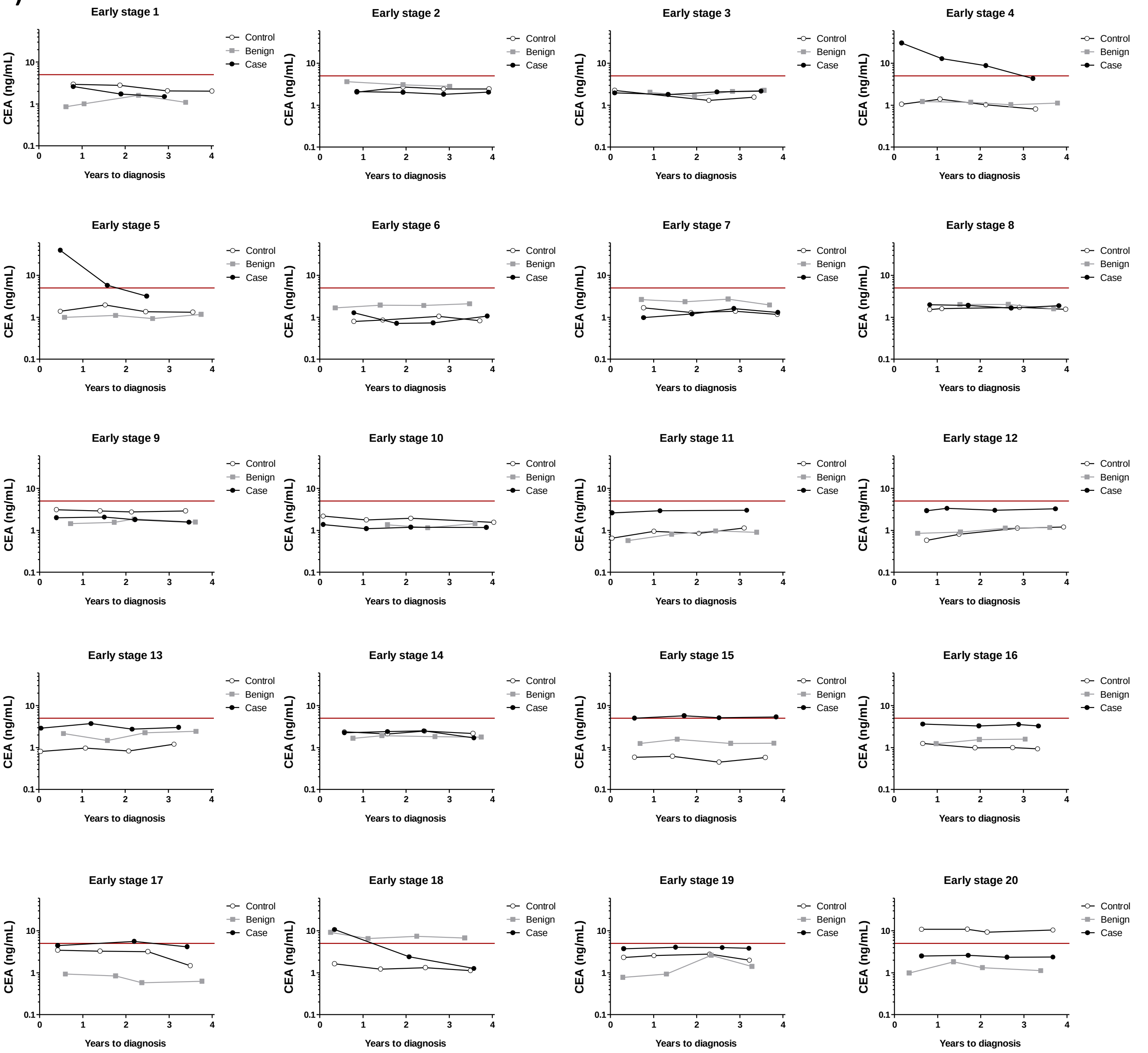

(B)

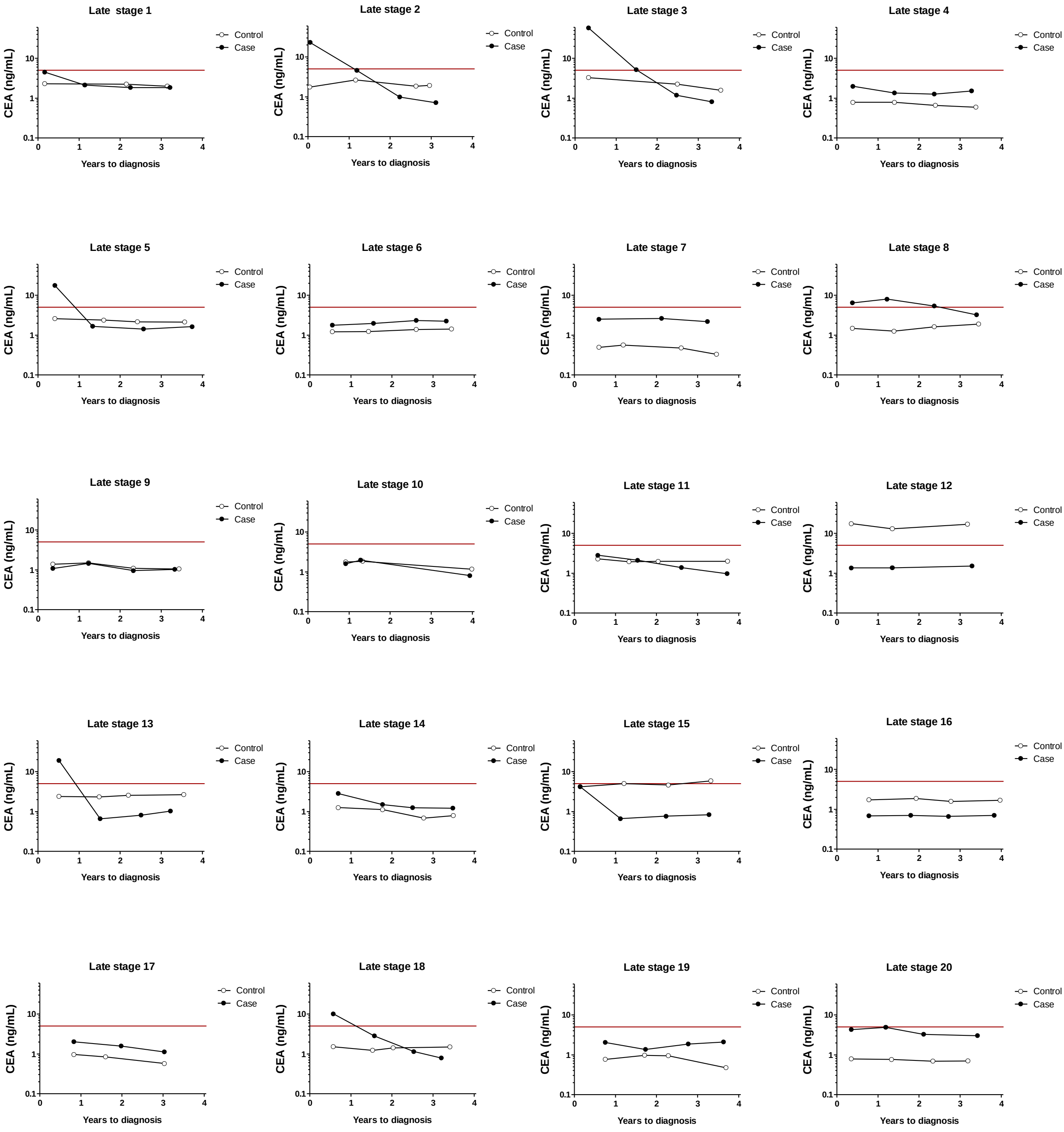

Late stage 1
Late stage 2
Late stage 3
Late stage 4
Late stage 5
Late stage 6
Late stage 7
Late stage 8
Late stage 9
Late stage 10
Late stage 11
Late stage 12
Late stage 13
Late stage 14
Late stage 15
Late stage 16
Late stage 17
Late stage 18
Late stage 19
Late stage 20
CEA (ng/mL)
Years to diagnosis
Control
Case
0.1
1
10
0
1
2
3
4
